# The drivers of avian‐haemosporidian prevalence in tropical lowland forests of New Guinea in three dimensions

**DOI:** 10.1002/ece3.8497

**Published:** 2022-02-14

**Authors:** Celia Vinagre‐Izquierdo, Kasun H. Bodawatta, Kryštof Chmel, Justinn Renelies‐Hamilton, Luda Paul, Pavel Munclinger, Michael Poulsen, Knud A. Jønsson

**Affiliations:** ^1^ Natural History Museum of Denmark University of Copenhagen Copenhagen Denmark; ^2^ Section for Ecology and Evolution Department of Biology University of Copenhagen Copenhagen Denmark; ^3^ Conservation and Evolutionary Genetics Group Estación Biológica de Doñana – CSIC Sevilla Spain; ^4^ Department of Zoology Faculty of Sciences University of South Bohemia České Budějovice Czech Republic; ^5^ Biology Centre Czech Academy of Sciences České Budějovice Czech Republic; ^6^ New Guinea Binatang Research Centre Madang Papua New Guinea; ^7^ Department of Zoology Faculty of Science Charles University Prague Czech Republic

**Keywords:** forest cover, *Haemoproteus*, host–parasite networks, Normalized Difference Vegetation Index (NDVI), *Plasmodium*, vertical stratification

## Abstract

Haemosporidians are among the most common parasites of birds and often negatively impact host fitness. A multitude of biotic and abiotic factors influence these associations, but the magnitude of these factors can differ by spatial scales (i.e., local, regional and global). Consequently, to better understand global and regional drivers of avian‐haemosporidian associations, it is key to investigate these associations at smaller (local) spatial scales. Thus, here, we explore the effect of abiotic variables (e.g., temperature, forest structure, and anthropogenic disturbances) on haemosporidian prevalence and host–parasite networks on a horizontal spatial scale, comparing four fragmented forests and five localities within a continuous forest in Papua New Guinea. Additionally, we investigate if prevalence and host–parasite networks differ between the canopy and the understory (vertical stratification) in one forest patch. We found that the majority of Haemosporidian infections were caused by the genus *Haemoproteus* and that avian‐haemosporidian networks were more specialized in continuous forests. At the community level, only forest greenness was negatively associated with *Haemoproteus* infections, while the effects of abiotic variables on parasite prevalence differed between bird species. *Haemoproteus* prevalence levels were significantly higher in the canopy, and an opposite trend was observed for *Plasmodium*. This implies that birds experience distinct parasite pressures depending on the stratum they inhabit, likely driven by vector community differences. These three‐dimensional spatial analyses of avian‐haemosporidians at horizontal and vertical scales suggest that the effect of abiotic variables on haemosporidian infections are species specific, so that factors influencing community‐level infections are primarily driven by host community composition.

## Graphical abstract

Abiotic and biotic factors in combination influence host–parasite associations and here we studied tropical avian‐haemosporidians associations at local spatial scales to help understand the effects of natural and anthropogenic factors on parasite prevalence and specificity. Parasite prevalence levels of different host species are affected by different biotic and abiotic factors and host species‐specific effects in combination influence the driverse of host community‐level prevelances. Furthermore, forest type and forest strata can alter host–parasite associations, where fragmented forests favor more generalist parasites and the infection levels of different parasite genera differ between understory and canopy strata.

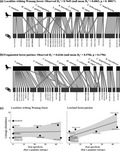

## 1 INTRODUCTION

Parasites are ubiquitous, diverse, and play major ecological roles in terrestrial and aquatic ecosystems (García del Río et al., 2020; Poulin, 1999), where they are a prominent selective force that influences fitness, distribution, and evolution of hosts (Poulin, 1998). Haemosporidians (Phylum Apicomplexa) are blood parasites transmitted by dipteran vectors and are among the most common parasites in vertebrates (Soares et al., 2017), including in birds (Hellgren et al., 2009; Valkiūnas, 2005). Infections in birds generally impact host fitness negatively (Atkinson, 2009; LaPointe et al., 2012; Rivero & Gandon, 2018) and the introduction of haemosporidians to naïve bird communities (e.g., on previously isolated islands) can have dramatic consequences and even lead to population collapses or species extinctions (Ewen et al., 2012; Freed et al., 2005).

Associations between birds, haemosporidian parasites, and dipteran vectors are governed by both biotic (e.g., host availability and density) and abiotic (e.g., temperature and precipitation) factors (Chapa‐Vargas et al., 2020), in addition to anthropogenic alterations, such as deforestation and habitat degradation (Atoyan et al., 2018; Chasar et al., 2009; Marzal et al., 2015; Olsson‐Pons et al., 2015; Sehgal, 2010). Temperature, humidity, precipitation and proximity to water appear to be the most important environmental variables influencing avian‐haemosporidian interactions on regional and global scales (Illera et al., 2017; Jones et al., 2013; Mendenhall et al., 2013; Padilla et al., 2017; Villar Couto et al., 2019). However, prevalence varies enormously between years (Bensch et al., 2007; Lachish et al., 2011; Ricklefs et al., 2005) and seasons (Cosgrove et al., 2008; Hernández‐Lara et al., 2017), even for climatically similar localities on smaller spatial scales (Hernández‐Lara et al., 2017; Knowles et al., 2014; Wood et al., 2007). Vector communities may also differ between forest canopy and understory (Bates, 1944; Brant et al., 2016; Chakarov et al., 2020; Clements, 1999), implying that parasite transmission may differ significantly within a locality. Prevalence at larger scales may thus be driven by a combination of small‐scale local variation in biotic and abiotic factors.

**FIGURE 1:**
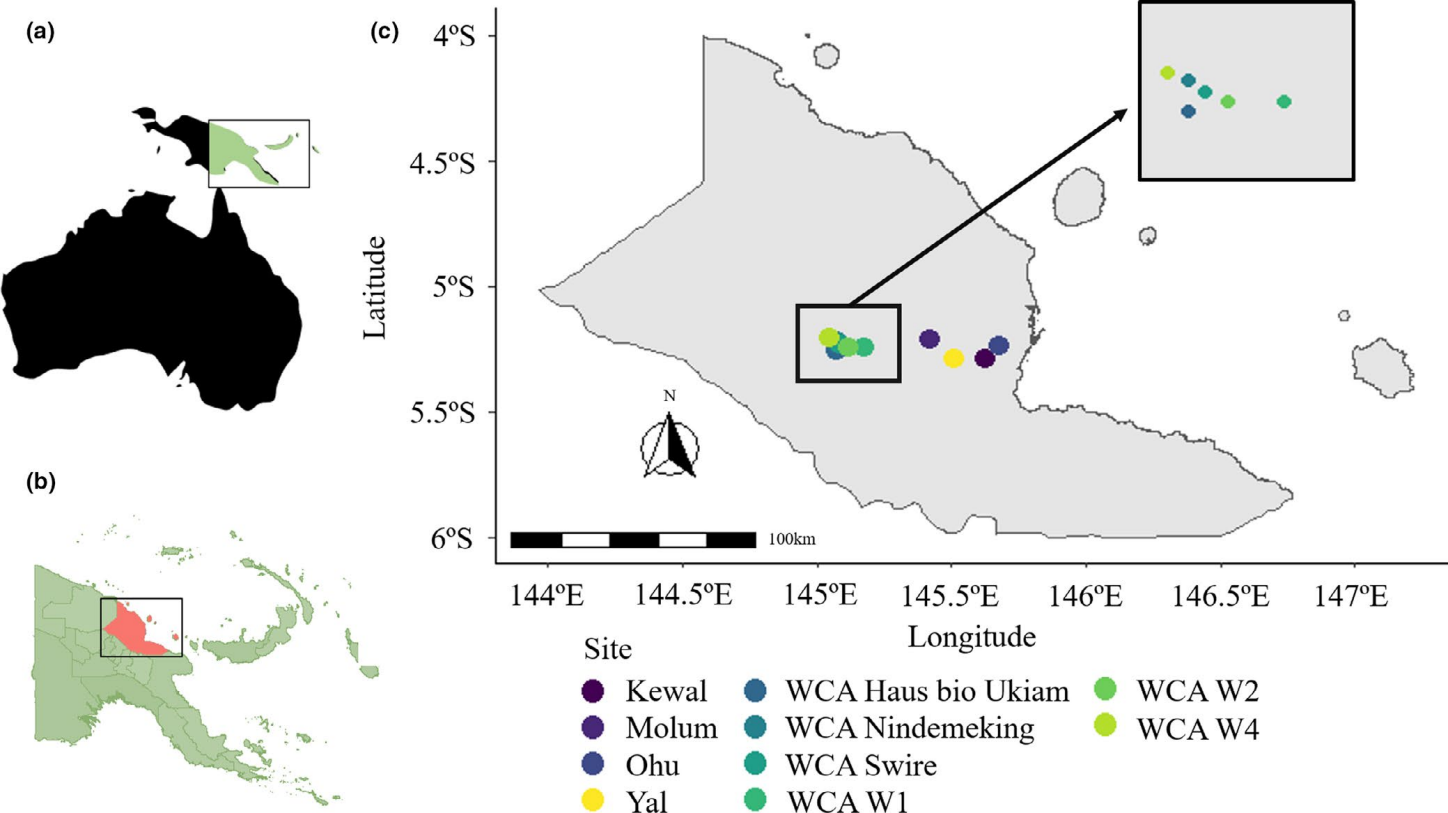
Location of sampling sites in northern Papua New Guinea (PNG). Map depicting (a) the location of PNG in Oceania, and (b) the location of the Madang province in Northern PNG. (c) Map of the Madang province indicating the 10 sampling sites (9 in 2015 and 1 in 2013) located on an east to west axis. Code names starting with WCA are within the continuous Wanang Conservation Area. The WCA Swire locality was only sampled in 2013

A first step towards understanding what determines local, regional, and ultimately global avian‐haemosporidian prevalence patterns is to decipher the factors that govern prevalence in individual host species at local spatial scales. We address this by investigating haemosporidian prevalence, host specificity, and host–parasite networks of lowland bird species in multiple forest localities in close geographic proximity in Papua New Guinea. We sampled 10 abundant bird species along an east–west axis (spanning ~70 km) in 4 fragmented forest patches (~48 km apart) as well as 5 localities within a single continuous primary forest (~14 km apart) (Figure 1). Additionally, in one locality within the continuous primary forest (Figure 1), we tested the effect of vertical stratification by comparing understory and canopy parasite prevalence and host–parasite network structures in four confamilial host species pairs.

## 2 MATERIALS AND METHODS

### 2.1 Field sites and sample collection

All samples were collected in Madang Province of northern Papua New Guinea (PNG). In 2015, samples were collected across nine sampling sites (on an east to west axis from the coast to inland), representing four small forest fragments close to human settlements (Ohu Village, Kewal Village, Yal Village, and Molum Village) and five localities within the large continuous forest of the Wanang Conservation Area (WCA) (WCA W1, WCA W2, WCA_Haus_bio_Ukiam, WCA_Nindemekin, and WCA W4) (Figure 1). We focused on 10 common lowland species (*Arses insularis*, *Ceyx solitarius*, *Colluricincla megarhyncha*, *Meliphaga analoga*, *Melanocharis nigra*, *Pitohui kirhocephalus*, *Rhipidura leucothorax*, *Symposiachrus guttula*, *Tanysiptera galatea*, and *Toxorhamphus novaeguineae*) of which a total of 276 individuals were sampled. The birds were all captured using standard mist nets (~3 m height from the ground) (Table S1).

To explore the effect of vertical stratification on avian‐haemosporidian associations, we captured birds from the forest floor to 27 m above ground in 2013 at the Swire Station locality within WCA (Figure 1) using stacked mist nets (for details see Chmel et al., 2016). Stacked mist nets were only used in 2013 due to resource limitations. After the investigation of average capture heights and removal of species for which we had less than five individuals, we identified the following four pairs of confamilial understory and canopy species: *Symposiachrus guttula* and *Monarcha chrysomela* [family: Monarchidae—Monarch flycatchers], *Chalcophaps stephani* and *Ptilinopus magnificus* [Columbidae—Pigeons], *M*. *analoga* and *Xanthotis flaviventer* [Meliphagidae—Honeyeaters], and *T*. *galatea* and *Dacelo gaudichaud* [Alcedinidae—Kingfishers]) (in total, 135 individuals) (Figure S1, Table S2).

Body mass and tarsus length was measured for all individuals sampled, and 10–20 µl of blood was obtained from the brachial artery and stored in 70% ethanol until DNA extractions. To test for sex‐specific differences, we sexed individuals using PCRs with the primers 2550F and 2718R for Passeriformes and Columbiformes, and p2 and p8 for Coraciiformes (Fridolfsson & Ellegren, 1999). Heterogametic females and homogametic males were distinguished through visualization of PCR products on a 2% agarose gel.

### 2.2 Molecular identification of haemosporidians

DNA was extracted using the Qiagen DNeasy^®^ blood and tissue kit (Hilden, Germany), following the manufacturer's guidelines, with a prolonged incubation period (approximately 12 h at 56°C). Haemosporidians were identified through nested PCRs with slight modifications to a well‐established protocol (Bensch et al., 2000; Hellgren et al., 2004). The initial PCRs were conducted in triplicates using HaemNF1 (5′‐CATATATTAAGAGAAITATGGAG‐3′) and HaemNR3 (5′‐ATAGAAAGATAAGAAATACCATTC‐3′) primers and the PCR master mix contained a total volume of 25 μl per sample (12.5 μl of VWR RedTaq polymerase^®^, 1 μl of 10 mM concentration of each primer, 8.5 μl of autoclaved MilliQ water, and 2 μl of the DNA template). These PCRs were conducted under an initial step of 3 min at 94°C and 20 cycles of 30 s at 94°C, 30 s at 50°C, 45 s at 72°C, and 10 min at 72°C. We then proceeded with the second PCRs targeting specific haemosporidian genera (*Haemoproteus* and *Plasmodium)*, using HaemR2 (5′‐GCATTATCTGGATGTGATAATGGT‐3′) and HaemF (5′‐ATGGTGCTTTCGATATGCATG‐3′) primers (Hellgren et al., 2004). We did not investigate *Leucocytozoon* parasites due to their low prevalence in New Guinea (Bodawatta et al., 2020). The second PCR was set up using 10 μl of Qiagen multiplex master mix (Hilden, Germany), 1 μl of 10 mM concentration of each primer, and 8 μl of 10× diluted product from the first PCR. The second PCR was conducted with an initial step of 3 min at 94°C and 35 cycles of 30 s at 94°C, 30 s at 50°C, 45 s at 72°C, and 10 min at 72°C. Every PCR round contained a positive control and a negative control for every 16 samples. Final PCR products were visualized on a 2% agarose gel containing GelGreen^®^ stain at 90 V for approximately 1 h.

Positive PCR products were cleaned using PureIT ExoZAP PCR CleanUp (Amplicon, Odense, Denmark) and subsequently sequenced in a Sanger platform at Eurofins Denmark (Glostrup, Denmark) for the forward primer (HaemF). Samples with short (<479 base pairs [bp]) sequences were also sequenced for the reverse primer (HaemR2). Sequences were aligned using Geneious Prime v4.8.5, and mismatches were checked manually. Aligned sequences were then compared to the MalAvi database of avian malaria parasites and related haemosporidians (Bensch et al., 2009) using the *malaviR* v0.2.0 package in R (Vincenzo et al., 2017). Sequences that matched reference lineages in MalAvi with less than 98% (Bensch et al., 2000; Ricklefs & Fallon, 2002; Waldenström et al., 2004) were considered novel lineages.

### 2.3 Host–parasite networks, lineage specificity, and host phylogeny

To explore host–parasite network structures in different sampling localities we calculated the network‐level specificity index (H_2_′) for bird–haemosporidian communities using the R package *bipartite* v2.15 (Dormann et al., 2008). An H_2_′ index close to 1 indicates specialized host–parasite communities with more one‐to‐one interactions between host species and parasite lineages, while indices closer to 0 indicate more generalized networks (Blüthgen et al., 2006). We then compared the observed network specificity values with specificities expected by chance through generating 1,000 random networks, to investigate whether observed values deviate significantly from network specificities expected by chance. We also investigated haemosporidian lineage‐level specificity on the most common lineages (infecting >2 individuals) between the continuous forest and the fragmented forest patches (combining all localities within each category). We used the threshold of >2 individuals as the majority of our haemosporidian lineages only infected one bird species. We calculated specificity for each lineage using Rao's quadratic entropy, while incorporating phylogenetic distances among host species (accounting for the importance of host evolutionary histories on haemosporidian specificity levels) using the raoD function in the R package *picante* v1.8.2 (Kembel et al., 2010). Higher Rao values indicate more generalists while lower values indicate more specialist lineages (Ellis et al., 2020).

We generated a host species phylogeny using a concatenated alignment of three mitochondrial (NADH dehydrogenase 2: ND2, NADH dehydrogenase 3: ND3, and Cytochrome b: cytb) and three nuclear (Myoglobin intron 2: Myo2, Glyceraldehyde‐3‐Phosphate Dehydrogenase intron 11: GAPDH, Ornithine decarboxylase introns 6 and 7: ODC) genes, sourced from GenBank (Table S3) using BEAST v1.8.4 (Drummond et al., 2012). We applied the General Time Reversible nucleotide substitution model to the concatenated dataset and ran the analysis for 100 million generations using a relaxed uncorrelated lognormal distribution for the molecular clock model, and assuming a birth–death speciation process as a tree prior. Convergence diagnostics were assessed in Tracer v1.6 (Suchard et al., 2018), by determining the effective sample sizes and mean distribution values. The final output tree was summarized in TreeAnnotator v1.8.3 (Rambaut & Drummond, 2015) as a maximum clade credibility (MCC) tree after discarding the first 10 million generations as burn‐in.

### 2.4 Environmental data

Environmental variables for individual sampling localities (e.g., maximum and minimum temperature, elevation, and distance to large water bodies [rivers and the sea]) were gathered from online databases (see below). We used the distance to rivers as a proxy for habitat availability for vectors, but we do acknowledge that this parameter is suboptimal to fully understand the habitat availability for vectors, as vectors can breed in small water pools, such as water retained in tree stumps and bromeliads. Nevertheless, this index still provides an indication of water availability in the area. Furthermore, we collected metadata related to human activities such as vegetation type (e.g., farmlands, forests) and Normalized Difference Vegetation Index (NDVI: a proxy for forest greenness) (Grace & Gates, 1982), and distance to the closest roads for every locality. Raster layers for each variable were gathered from Diva‐GIS v7.5 (https://www.diva‐gis.org/Data), FreeMapTools (https://www.freemaptools.com/), Humanitarian Data Exchange v1.52.9 (https://data.humdata.org/), GeoNetwork – FAO (http://www.fao.org/geonetwork/), Copernicus Global Land Service (https://land.copernicus.eu/global/products/ndvi) and CHELSA databases (Beck et al., 2020; Karger et al., 2017, 2020; https://chelsa‐climate.org/). Raster layers and shapefiles were uploaded to QGIS v3.14.0 (QGIS Geographic Information System. QGIS Association, 2016) and to extract values for each locality, one vector file with the coordinates for each locality was created (Figures S2 and S3). Raster layers for abiotic variables were combined with the Merge tool from the GDAL package (Qin & Zhu, 2020), and mean values of every locality from all the layers were extracted with the Point Sampling Tool Plugin v0.5.3 (Jurgiel, 2020).

We used NDVI to estimate forest greenness as a proxy for forest structure. NDVI has been used extensively to evaluate forest structure (Grace & Gates, 1982), yet we acknowledge the inherent limitations (e.g., not capturing the changes in forest interior) of this measure. Nonetheless, NDVI provides a normalized value for forest greenness that is comparable across study sites and even between studies. NDVI was calculated using the following equation NDVI = (NIR − RED)/(NIR + RED), where NIR is the near‐infrared and RED the visible band (Myneni et al., 1995). It measures the degree of absorption by chlorophyll in red wavelengths (Myneni et al., 1995), the index values fall between −1 and 1, with values around −1 representing clouds and water, values around 0 representing bare soil, and values close to 1 representing forested areas with maximum greenness (i.e., forest cover) (Atoyan et al., 2018). For environmental variables that had a low resolution for the exact GPS coordinate, we used the value of the adjacent pixel (<800 m from the original point) to that locality. We used the NNJoin Plugin v3.1.3 (Tveite, 2019), to calculate nearest neighbor relationships (Eppstein et al., 1997) from each locality to rivers, roads, and the sea (Tables S1 and S4; Figures S2 and S3).

### 2.5 Statistical analyses

Statistical analyses were conducted using R v3.6.3 (R Core Team, 2020). Haemosporidian parasites genera (*Haemoproteus* spp. and *Plasmodium* spp.) were analyzed separately, using binomial (presence/absence of parasites) generalized linear models (GLMs), and phylogenetic generalized linear mixed models (PGLMMs). Due to very low prevalence (<5%) of *Plasmodium*, only *Haemoproteus* was included in the analyses in the 2015 dataset (Table 1; Table S1). Furthermore, because *M*. *nigra* and *P*. *kirhocephalus* had high parasite prevalence in all localities, with little to no variation (99%–100%), they were excluded from the linear models (Table 1).

First, we examined the collinearity of abiotic variables using Pearson's correlation tests with the function *ggpairs* from the R package *GGally* v2.0.0 (Schloerke et al., 2019), and found that multiple variables that were significantly correlated with each other (Figure S4). Thus, for the final analyses, we only included variables that were not collinear (NDVI, Minimum temperature, Distance to roads). Although, NDVI was positively, yet nonsignificantly, correlated with the vegetation type (Pearson correlation: *r* = .6270, *p* = .1001), we chose to include NDVI rather than vegetation type due to NDVI being more accurate. We further checked spatial autocorrelation of environmental variables considering latitude and longitude of the sampling localities using Monte Carlo tests with the function *mantel*.*rtest* from the R package *ade4* v1.7–18 (Thioulouse et al., 2018) and found no autocorrelations (NDVI: *p* = .5138, Mantel *r* = −.0527; Minimum temperature: *p* = .6706, Mantel *r* = −.0943; Distance to roads: *p* = .0622, Mantel *r* = .2916; based on 9999 replicates).

We performed both community‐level and species‐level models to investigate the effect of the abiotic variables on *Haemoproteus* infections (as the dependent variable) in 2015. In the community level, we conducted PGLMM using the pglmm function in *phyr* v1.1.0. package to account for host phylogenetic relationship (Li et al., 2020). Here we included host species, site, sex, NDVI, distance to the roads, and minimum temperature as the independent variables and the distance to sea as a random effect to control for the spatial arrangement of the sampling sites (our sampling sites are located in an east–west spatial scale from the sea: Figure 1). Following the guidelines in Crawley (2013), we did model selection procedures for the PGLMMs, and variables that were not significant were eliminated from the model, resulting in a final model which considered species and NDVI as variables with significant influence. We used type‐II analysis of variance (ANOVAs) from the *car* package v3.0.9 (Fox & Weisberg, 2019) to obtain the *p*‐values for the variables. The species‐level models (separate GLM per species) were conducted similarly to the community‐level analyses, without host species. To investigate the effect of vertical stratification (data gathered in 2013), we used separate models for *Haemoproteus* and *Plasmodium* infections with the stratum (understory or canopy), sex, and family as independent variables.

## 3 RESULTS

### 3.1 Haemosporidian prevalence and lineage diversity

Overall, 185 of the 276 bird individuals (67.0%) were infected with haemosporidian parasites in 2015 (61.2% with *Haemoproteus* and 4.7% with *Plasmodium*) across the 9 sampling sites, while 101 of the 135 tested individuals were infected in 2013 at the WCA_Swire locality (67.9% *Haemoproteus* and 7.5% *Plasmodium*). Haemosporidian sequences (at least 479 bp) acquired in 2015 belonged to 41 lineages, while 35 belonging to *Haemoproteus* and 6 to *Plasmodium*. From WCA_Swire (2013), we acquired 37 unique lineages (24 *Haemoproteus* and 13 *Plasmodium*). All the parasite lineages match to known lineages in MalAvi database.

Due to the low prevalence of *Plasmodium* in the 2015 dataset, we were unable to investigate the influence of abiotic variables on *Plasmodium* infections, so the subsequent analyses were only conducted on *Haemoproteus*. *Haemoproteus* prevalence differed significantly between host species (binomial GLM: *LR χ* ^2^ = 65.18, df = 8, *p* < .0001; Figure S5) and not between locations (binomial GLM: *LR χ* ^2^ = 12.18, df = 8, *p* = .1431; Figure S6) suggesting that some bird species are more susceptible to infections than others. However, prevalence did not differ between the sexes (binomial GLM: *LR χ* ^2^ = 2.032, df = 3, *p* = .5658). The strong host species effect further supported conducting statistical analyses on both host community and species levels.

### 3.2 More specialized host–parasite networks in localities within continuous forest

**FIGURE 2:**
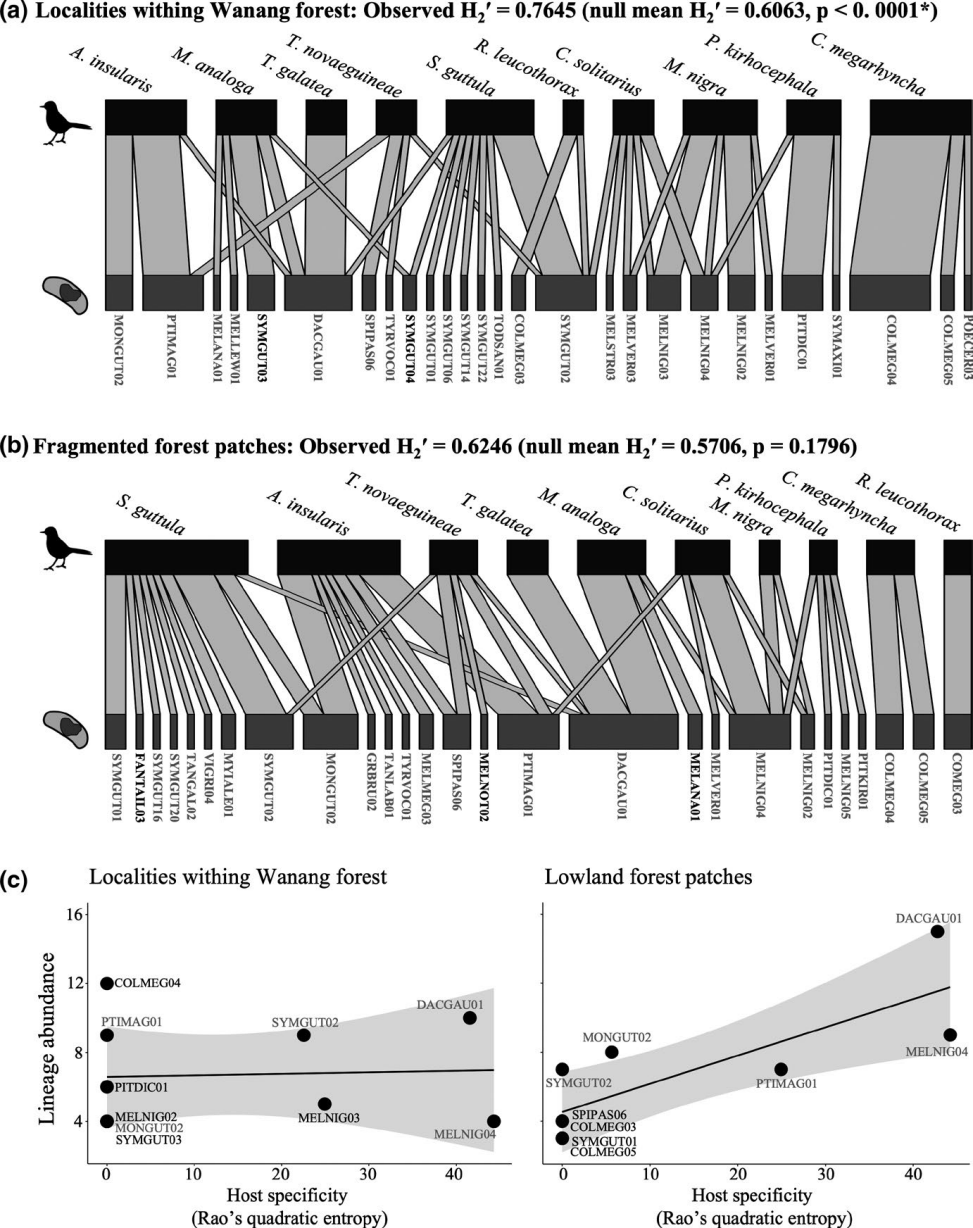
Bird–haemosporidian networks and host specificity in different forest categories (continuous vs. fragmented). Networks indicate combined host–parasite communities of the five localities within Wanang Conservation Area (a) and four fragmented forest patches (b). An H_2_′ index closer to 1 indicates that host–parasite communities are more specialized (many one‐to‐one associations), while highly generalized networks have H_2_′ indices closer to 0. Observed H_2_′ and the average H_2_′ acquired from 1,000 null models are given within parentheses. Asterisks indicate significantly different observed H_2_′ values compared to the null expectation. *Haemoproteus* lineage names are given in gray and *Plasmodium* lineage names are in black. c. Association between host specificity of the most abundant haemosporidian lineages (Rao's quadratic entropy) and the lineage abundance in the continuous forest and fragmented forests. Parasite lineages found in both forest categories are given in gray. Large Rao values indicate generalist lineages while small values indicate specialist lineages. Host specificity was only calculated for parasite lineages that infected more than two bird individuals

Host–parasite network structure was more specialized than expected by chance throughout continuous forest localities (H_2_′ = 0.7645, null mean_1,000 random iterations_: 0.6063, *p* < .0001; Figure 2a), while network structure of fragmented forests displayed more random associations (H_2_′ = 0.6246, null mean_1,000 random iterations_: 0.5706, *p* = .1796; Figure 2b). This was consistent across individual localities, except for two within the continuous forest (Figures S7 and S8). This indicates that avian‐haemosporidian networks within the undisturbed forests are more specialized than those of fragmented forests. Host specificity of lineages that infected more than two individual hosts was significantly positively associated with lineage abundance in fragmented forests (lm: *F* = 13.58, *R* ^2^ = .6113, *p* = .0078), but not in the continuous forest (lm: *F* = 0.0233, *R* ^2^ = .1217, *p* = .8824) (Figure 2C).

### 3.3 Species‐specific effects of abiotic factors on *Haemoproteus* prevalence between localities

**FIGURE 3:**
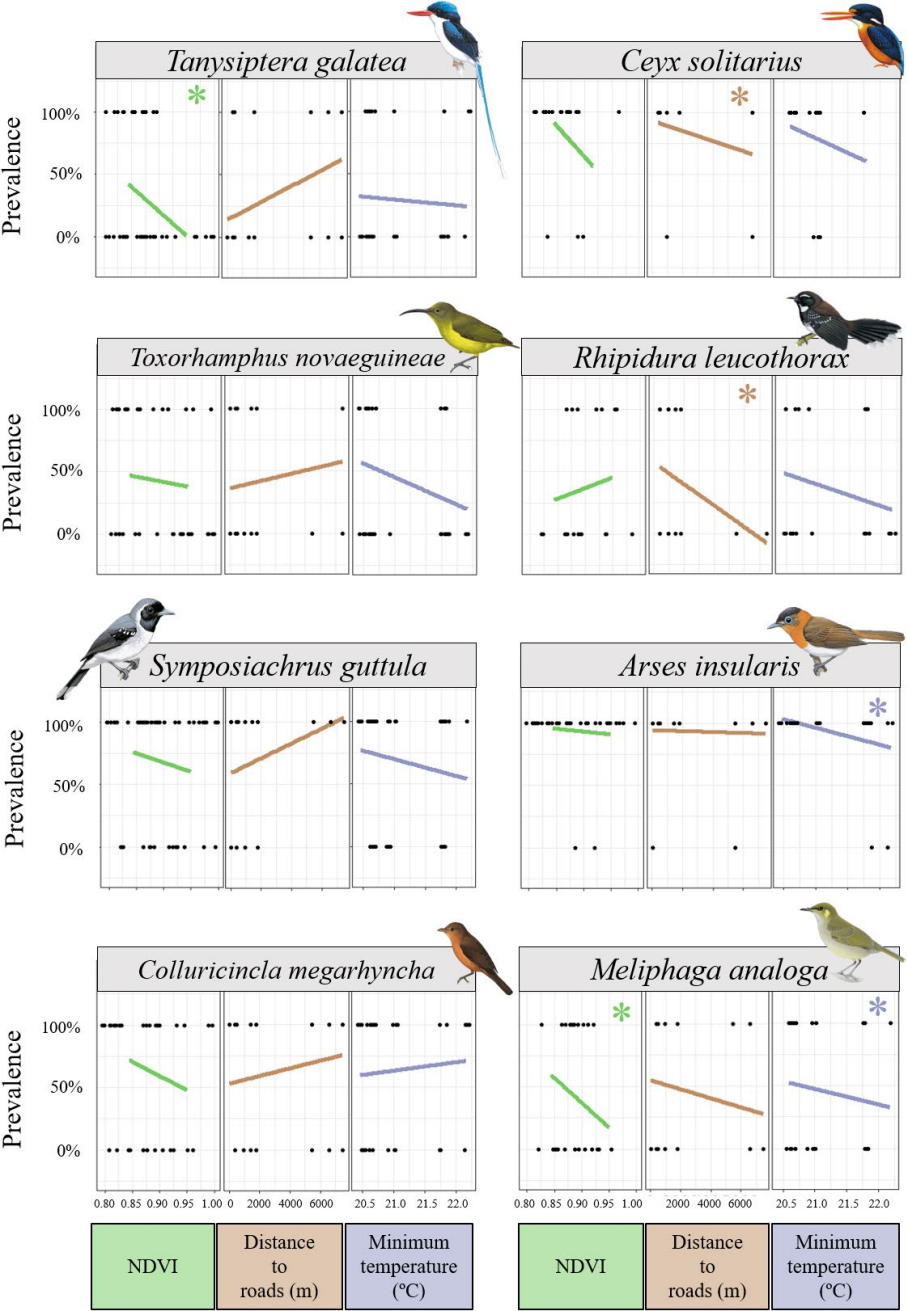
Associations of *Haemoproteus* prevalence with minimum temperature (°C), distance to roads (m) and NDVI across all sampling sites for all species sampled in 2015. *Pitohui kirhocephalus* and *Melanocharis nigra* were not included in these analyses as all individuals were infected. Dots represent infected (100%) or noninfected (0%) individuals and lines (linear model estimates) show the prevalence changes associated with each variable. Significant effects from the GLMs are indicated with asterisks. Bird illustrations were acquired from Lynx Edicions©

Despite the significant effect of locality on *Haemoproteus* prevalence, there was no significant difference between continuous and fragmented forests (binomial GLM: *LR χ* ^2^ = 0.0646, df = 1, *p* = .7993; Figure S9). We found 27 unique haemosporidian haplotypes in the forest fragments and 26 in the continuous forest (Figure S10). At the bird community level, NDVI was the only significant predictor of *Haemoproteus* prevalence, which decreased with increasing NDVI (binomial PGLMM: *Std*.*error* = 3.381, Zscore = −2.480, *p* = .0131, Figure S11). However, species‐level analyses (Table 1) revealed significant effects of several predictors on *Haemoproteus* prevalence, and these effects were host species specific (Table 1, Figure 3). Increased NDVI affected *Haemoproteus* prevalence negatively for all species except in *R*. *leucothorax*; yet, the association was only significant for *T*. *galatea* and *M*. *analoga* (Table 1, Figure 3). On the other hand, *C*. *solitarius*, *R*. *leucothorax*, and *M*. *analoga* bore higher *Haemoproteus* prevalence the closer they were to roads (Table 1, Figure 3). Minimum temperature negatively affected parasite prevalence for all host species, except *C*. *megarhyncha*; however, this was only significant for *A*. *insularis* (Table 1, Figure 3).

**TABLE 1:** Species‐level effects based on binomial GLM analyses of abiotic variables (NDVI and minimum temperature) and distance to roads (proxy for anthropogenic disturbance) on *Haemoproteus* prevalence in 2015

Species	Dependent variable	Independent variable	LR *χ* ^2^	df	Pr (>*χ* ^2^)
*Arses insularis*	*Haemoproteus*	NDVI	1	1	1
		Minimum temperature	6.279	1	0.0122*
		Distance to roads	−0	1	1
*Ceyx solitarius*	*Haemoproteus*	NDVI	3.587	1	0.0582
		Minimum temperature	1.694	1	0.1930
		Distance to roads	3.984	1	0.0459*
*Tanysiptera galatea*	*Haemoproteus*	NDVI	3.995	1	0.0456*
		Minimum temperature	0.8908	1	0.3453
		Distance to roads	1.563	1	0.2112
*Toxoramphus novaeguineae*	*Haemoproteus*	NDVI	0.2827	1	0.5950
		Minimum temperature	2.291	1	0.1302
		Distance to roads	0.3801	1	0.5376
*Rhipidura leucothorax*	*Haemoproteus*	NDVI	1.562	1	0.2113
		Minimum temperature	1.548	1	0.2135
		Distance to roads	6.2813	1	0.0122*
*Symposiachrus guttula*	*Haemoproteus*	NDVI	0.1047	1	0.7462
		Minimum temperature	0.1353	1	0.7130
		Distance to roads	3.265	1	0.0708
*Colluricincla megarhyncha*	*Haemoproteus*	NDVI	0.4502	1	0.5022
		Minimum temperature	0.0978	1	0.7545
		Distance to roads	0.1434	1	0.7050
*Meliphaga analoga*	*Haemoproteus*	NDVI	6.432	1	0.0112*
		Minimum temperature	1.802	1	0.1794
		Distance to roads	4.949	1	0.0261*

Note

Significant effects are marked with an asterisk (*).

### 3.4 Prevalence levels of haemosporidian genera differed by forest strata

**FIGURE 4:**
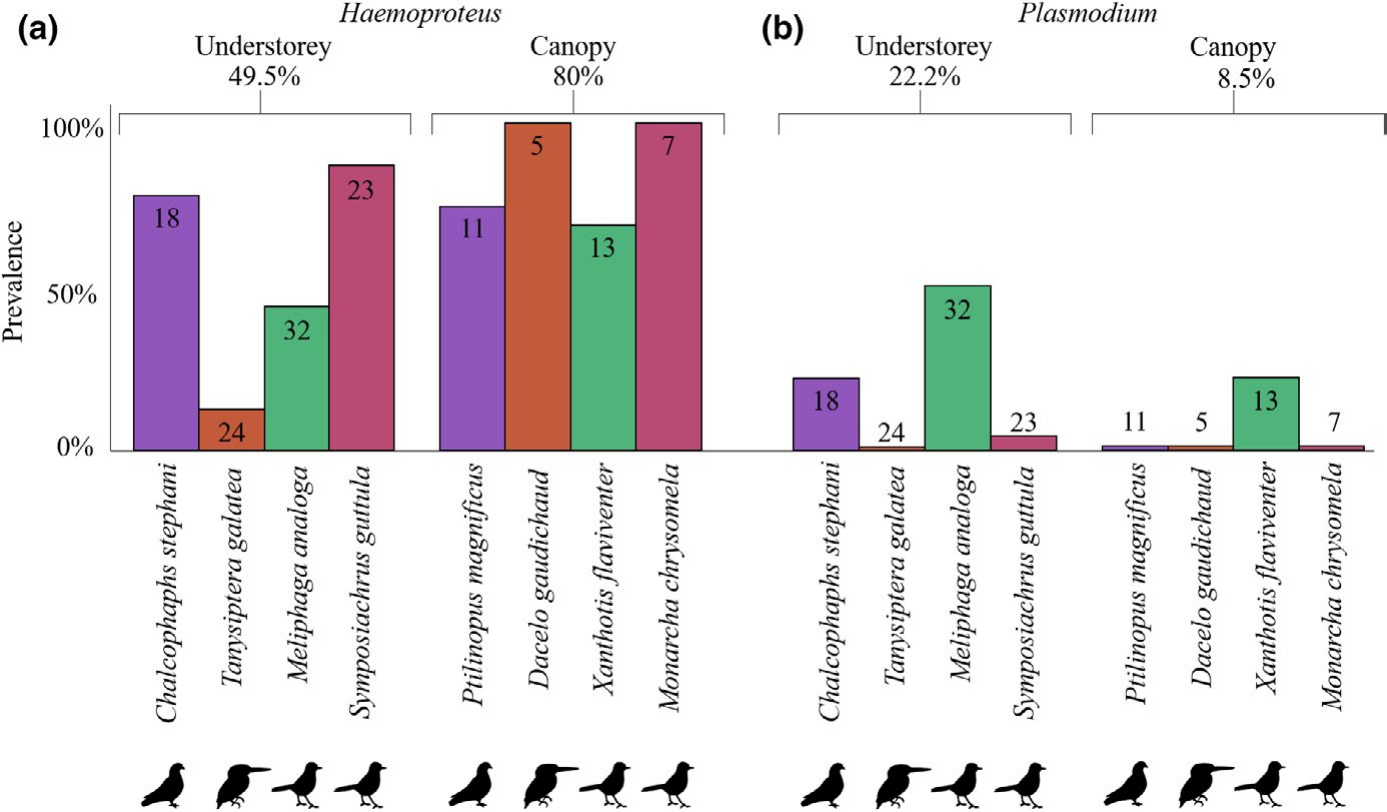
Haemosporidian prevalence of canopy and understory species. Bar graphs depict mean overall prevalence of (a) *Haemoproteus* and (b) *Plasmodium* across the species sampled in the canopy and in the understory at the WCA_Swire site in 2013. The number of samples is indicated at the top of the bars. Confamilial pairs are indicated with identical colors and silhouettes

We found significant differences in the overall prevalence of two haemosporidian genera between canopy (88.6%) and understory (71.1%) hosts (Figure 4a) in the 2013 dataset. *Haemoproteus* infections were significantly greater in the canopy (80.0%) compared to the understory (49.5%) (binomial GLM: *LR χ* ^2^ = 12.12, df = 1, *p* = .0005, Figure 4a), while *Plasmodium* prevalence, although overall low, was significantly higher in the understory (22.2%) than the canopy (8.5%) (binomial GLM: *LR χ* ^2^ = 9.340, df = 1, *p* = .0022, Figure 4a). Bird families were similarly infected between strata (binomial GLM: *LR χ* ^2^ = 3.568, df = 1, *p* = .3198) and so were different sexes (binomial GLM: *LR χ* ^2^ = 1.1407, df = 1, *p* = .5653). There were 23 unique haemosporidian haplotypes in the understory and 14 in the canopy, of which 17 and 12, respectively, were *Haemoproteus*. Of these lineages, only seven were shared between strata, indicating strata‐specific distribution of haemosporidian lineages (Figure S12). Host–parasite network structures of understory species (H_2_′ = 0.8364, null mean_1,000 random iterations_: 0.7447, *p* = .1291) and canopy (H_2_′ = 0.9067, null mean_1,000 random iterations_: 0.8424, *p* = .2757) revealed high network‐level specialization (H_2_′) however, these did not differ significantly from random expectations (Figures S13A,B). Interestingly, there were more specialist lineages (when considering lineages in >two individuals) in the canopy than the understory (Figure S13C), suggesting that the high prevalence observed in the canopy may be a result of the presence of more specialized lineages (Figure 4).

## 4 DISCUSSION

We investigated the influence of environmental and anthropogenic factors on avian‐haemosporidian (*Haemoproteus* and *Plasmodium*) parasite prevalence, distribution, specificity, and host–parasite network structures in tropical lowland birds at horizontal and vertical spatial scales. *Haemoproteus* was the most common parasite genus, aligning with previous work in Papua New Guinea (Bodawatta et al., 2020), who sampled at a site less than 25 km from our study sites. However, *Haemoproteus* prevalence was overall markedly lower in the previous study (~15%). The lowland study site sampled by Bodawatta et al. (2020) represents the lowest part of the Mount Wilhelm elevational gradient and includes the total bird community, while our localities are part of an extensive lowland area and only include 10 abundant bird species. Topographical differences and sampled avian communities of the two localities may thus at least in part explain the observed prevalence‐level differences. *Plasmodium* prevalences were low (5%) in the longitudinal study, consistent with findings by Bodawatta et al. (2020). This could be explained by hosts being less susceptible to this genus (Lima & Pérez‐Tris, 2020) or geographic variation in the distribution and density of *Plasmodium* vectors (Ferreira et al., 2020).

At the bird community level, NDVI (greenness) was the only variable that significantly influenced *Haemoproteus* prevalence. However, at the bird species level, the picture is less clear, with species‐specific effects of minimum temperature, distance to roads, and NDVI. Furthermore, we found vertical segregation in host–parasite interactions with higher prevalence in the canopy than the understory. *Haemoproteus* accounted for the majority of infections in both strata, but with a higher relative proportion in the canopy. Collectively, this not only suggests that specific vector communities may influence the transmission of particular malarial lineages but also that adaptation to particular ecological niches of a host species makes them differentially susceptible to pathogens.

### 4.1 More specialized host–parasite networks in undisturbed forests

We did not find significant differences in *Haemoproteus* prevalence between the localities within the continuous forest and the fragmented forests, which aligns with results from regional spatial scale studies in the Neotropics and the Afrotropics (Belo et al., 2011; Chasar et al., 2009; Loiseau et al., 2010; Rivero de Aguilar et al., 2018; Sebaio et al., 2012). However, our findings contrast a study from tropical Australia which found higher *Haemoproteus* prevalence in continuous than fragmented forest (Laurance et al., 2013). Higher prevalence levels in continuous forest have been speculated to be a result of higher vector abundances (Mangudo et al., 2017; Zhou et al., 2007). Thus, forest structure could indirectly affect parasite infection risk through influencing the vector abundances (Mangudo et al., 2017; Zhou et al., 2007), implying that vector sampling across forest types is needed to decipher their potential effects on prevalence levels between continuous and fragmented forests.

While prevalences did not differ between the continuous and fragmented forests, host–parasite network structures were notably different, where continuous forests harbored significantly more specialized networks than fragmented forests. The greater specialization in continuous forests could imply that undisturbed forests may provide more stable environments with higher host species richness (Bregman et al., 2014; Sam et al., 2014; Van Hoesel et al., 2020) that could lead to more specialized associations. Highly specialized avian‐haemosporidian networks has been observed before in an undisturbed tropical lowland bird community in Ecuador (Svensson‐Coelho et al., 2014). Fragmented forests, on the other hand, tend to favor generalist parasite lineages (driving observed random host–parasite network structures), which is evident by the observed association between host specificity of lineages and their abundances in the fragmented but not in the continuous forest. This aligns with the niche‐breadth hypothesis (Ellis et al., 2020; Pinheiro et al., 2016), predicting that generalist parasite lineages with broader host niches perform better in small forest patches than specialist lineages. The differences in lineage specificity and abundances in fragmented versus continuous forests may thus result from (i) changes in bird communities (abundances and densities) (Bodawatta et al., 2020; Fecchio, Bell, et al., 2019; Fecchio, Wells, et al., 2019), (ii) changes in the potential for competition between haemosporidian lineages (Bodawatta et al., 2020), and/or (iii) changes in environmental variables associated with forest fragmentation (Afrane et al., 2006).

### 4.2 Haemosporidian prevalence levels depict species‐specific responses to environmental and anthropogenic factors

Of the environmental variables, only increased NDVI (greenness) led to significantly reduced *Haemoproteus* prevalence, suggesting that minimum temperature and distance to roads do not significantly affect community‐level haemosporidian prevalence at local spatial scales. NDVI appears to be a good predictor for vector abundance and distribution (Roiz et al., 2015) and has been shown to be—in contrast to our findings—positively associated with *Haemoproteus* prevalence in seasonal temperate regions (Clark et al., 2020). However, our tropical localities had very high (0.8–1.0) NDVI with minor differences between sites, compared to studies in temperate regions (Fecchio et al., 2020; Ferraguti et al., 2018; Roiz et al., 2015). Thus, our results are not directly comparable with studies conducted in temperate regions but open the possibility of a nonlinear relationship between NDVI and parasite prevalence. These findings support the need for research on the effect of NDVI on *Haemoproteus* vector communities (i.e., biting midges) in tropical lowlands. We note that NDVI is significantly correlated with elevation above sea‐level and vegetation type, suggesting that the observed results could also be due to other factors that correlate with NDVI.

The species‐specific effect of different environmental variables on *Haemoproteus* prevalence (Figure 2) aligns with results from other studies on bird species from both temperate and tropical regions (Isaksson et al., 2013; Samuel et al., 2015; Santiago‐Alarcon et al., 2019; Van Hoesel et al., 2020). Despite the overall nonsignificant effect of distance to roads (a proxy for anthropogenic influence), it did significantly affect prevalence in three bird species, suggesting the potential for elevated infection levels with increased anthropogenic activity. The influence of anthropogenic activity on haemosporidian prevalence and their vectors has been documented for multiple bird species across geographical localities, showing either positive (Abella‐Medrano et al., 2015), negative (Chasar et al., 2009; Gonzalez‐Quevedo et al., 2014), or no (Sehgal, 2015) effects. This is consistent with our findings, as the magnitude of anthropogenic activity effects on prevalence differs from bird species to species.

Temperature tends to positively impact haemosporidian prevalence at a regional scale in both tropical and temperate regions (Chapa‐Vargas et al., 2020; Padilla et al., 2017; Sehgal, 2015; Zamora‐Vilchis et al., 2012). However, we found that increased minimum temperature (even minor differences, ~1.5°C) had a negative effect on *Haemoproteus* prevalence across multiple species (albeit only significantly for *A*. *insularis*) (Figure 2). Consensus on the effect of temperature on parasite prevalence in birds in the Australo‐Papuan region is lacking, as studies have shown positive (Zamora‐Vilchis et al., 2012) or no (Bodawatta et al., 2020) effects. Areas with lower temperatures experience more rainfall in our study region, indicating potentially more vector breeding habitats (Lapointe et al., 2012; Sehgal, 2015) that could lead to higher vector abundances and increased prevalence. In summary, our findings imply that the sum of species‐specific responses to different environmental variables dictate community‐level effects of abiotic factors in tropical bird communities.

### 4.3 Higher prevalence and reduced diversity of haemosporidians in the canopy

Higher *Haemoproteus* prevalence in the canopy than the understory aligns with previous findings from the Afrotropics (Lutz et al., 2015). However, *Plasmodium* prevalence was higher in the understory than canopy, which may reflect higher mosquito abundances (*Plasmodium* vectors) at the forest floor. Our finding suggests that the pattern might be opposite for biting midge vectors of *Haemoproteus* that are conceivably higher in the canopy as they tend to prefer these sites to ground strata (Černý et al., 2011; Garvin & Greiner, 2003; Swanson & Adler, 2010; Swanson et al., 2012). Only 5 of the 32 haemosporidian lineages were shared between the strata, likely due to vertical segregation of vector species (Henry & Adkins, 1975), implying that investigations of canopy and understory bird communities in a locality is needed to fully capture host–vector–parasite diversity and associations.

## 5 CONCLUSIONS

Our results demonstrate that interactions between haemosporidian parasites and tropical avian hosts are influenced by a multitude of factors at different taxonomic levels and spatial scales. Forest structure influences associations between particular host species and parasite lineages, while parasite prevalence of a set of host species (the community) is driven by a combination of species‐specific environmental effects. Vertical separation within a single locality appears to expose avian hosts to markedly different parasite pressures, which is likely driven by vector communities. Taken together, these results emphasize the importance of investigating avian‐haemosporidian associations in space, for both individual host species and at the host community level. Finally, the species‐specific effects of environmental variables and vertical stratification on parasite prevalence accentuate that the factors driving these interactions can differ between global, regional, and local spatial scales.

## CONFLICT OF INTEREST

All the authors declare there is no competing interest related to the material of this manuscript.

## AUTHOR CONTRIBUTIONS

**Celia Vinagre‐Izquierdo:** Conceptualization (equal); data curation (lead); formal analysis (lead); investigation (equal); methodology (lead); software (lead); validation (equal); visualization (lead); writing – original draft (equal); writing – review and editing (lead). **Kasun H. Bodawatta:** Conceptualization (equal); data curation (supporting); formal analysis (supporting); investigation (equal); methodology (supporting); supervision (equal); visualization (supporting); writing – original draft (equal); writing – review and editing (equal). **Krystof Chmel:** Methodology (supporting); resources (lead); writing – review and editing (equal). **Justinn Renelies‐Hamilton:** Formal analysis (supporting); investigation (supporting); writing – review and editing (equal). **Luda Paul:** Resources (equal). **Pavel Munclinger:** Data curation (supporting); methodology (supporting); software (supporting); validation (equal); writing – review and editing (equal). **Michael Poulsen:** Conceptualization (equal); investigation (equal); project administration (lead); supervision (equal); writing – original draft (equal); writing – review and editing (equal). **Knud Andreas Jønsson:** Conceptualization (equal); funding acquisition (lead); investigation (equal); project administration (lead); supervision (equal); writing – original draft (equal); writing – review and editing (equal).

### OPEN RESEARCH BADGES

This article has been awarded Open Data, Open Materials Badges. All materials and data are publicly accessible via the Open Science Framework at https://doi.org/10.5281/zenodo.5776763.

## Supporting information

Fig S1‐S13

Table S1

Table S2

Table S3

Table S4

## Data Availability

New haemosporidian sequences are submitted to GenBank (ID: 2427653) and MalAvi. Complete datasets utilized in this study are given in Tables S1–S4.
