# Supplementary material for: The drivers of avian‐haemosporidian prevalence in tropical lowland forests of New Guinea in three dimensions
Source: Ecol Evol. 2022 Feb 14;12(2):e8497. doi: 10.1002/ece3.8497 (PMC8844478; doi:10.1002/ece3.8497)
Supplement: Supplementary file 1 — Fig S1‐S13 [file ECE3-12-e8497-s002.docx]

**
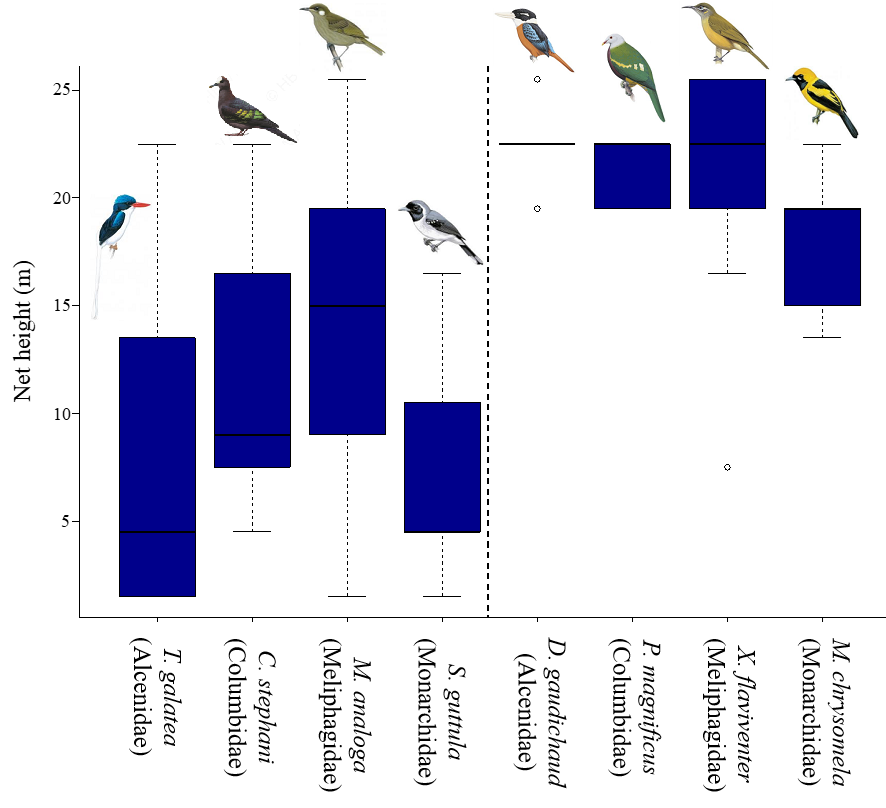
Figure S1. Box plots depict mean heights (± SD) of captured individuals of eight bird species included in the vertical stratification analyses.** Understorey species tend to occupy larger stratum ranges than canopy species. Mean heights of understorey species are below 15m above ground while the mean heights of canopy species are above ~18m.

**
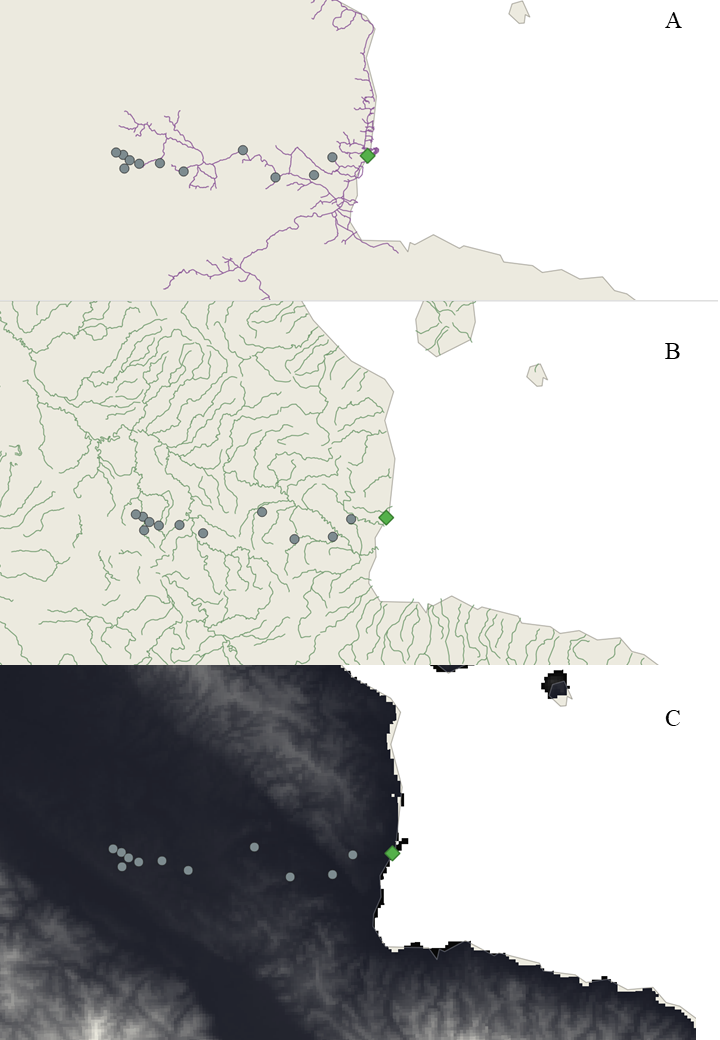
**

**Figure S2. GIS raster layers and locations from lowland PNG.** (A) Roads (purple lines) and locations, (B) distances to rivers and streams (green lines) and (C) elevation in lowland PNG. Darker colours in figure C indicate lower elevations. Grey dots indicate sampling locations and green square indicates Madang city. Images extracted from QGIS (version 3.14.0). Values for each location in supplementary table 4.

**
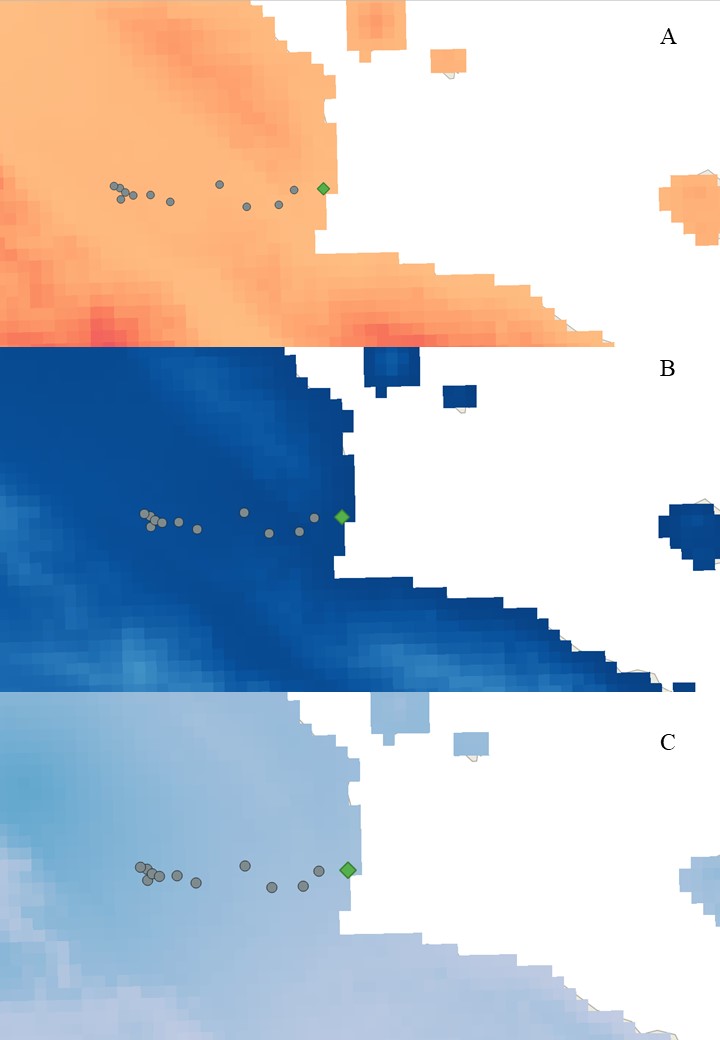
 Figure S3. GIS raster layers and locations from lowland PNG.** (A) Maximum temperature, (B) minimum temperature and (C) precipitation. Darker colours in every figure indicate higher values. Grey dots indicate sampling locations and green square indicates Madang city. Images extracted from QGIS (version 3.14.0). Values for each location in supplementary table 4.


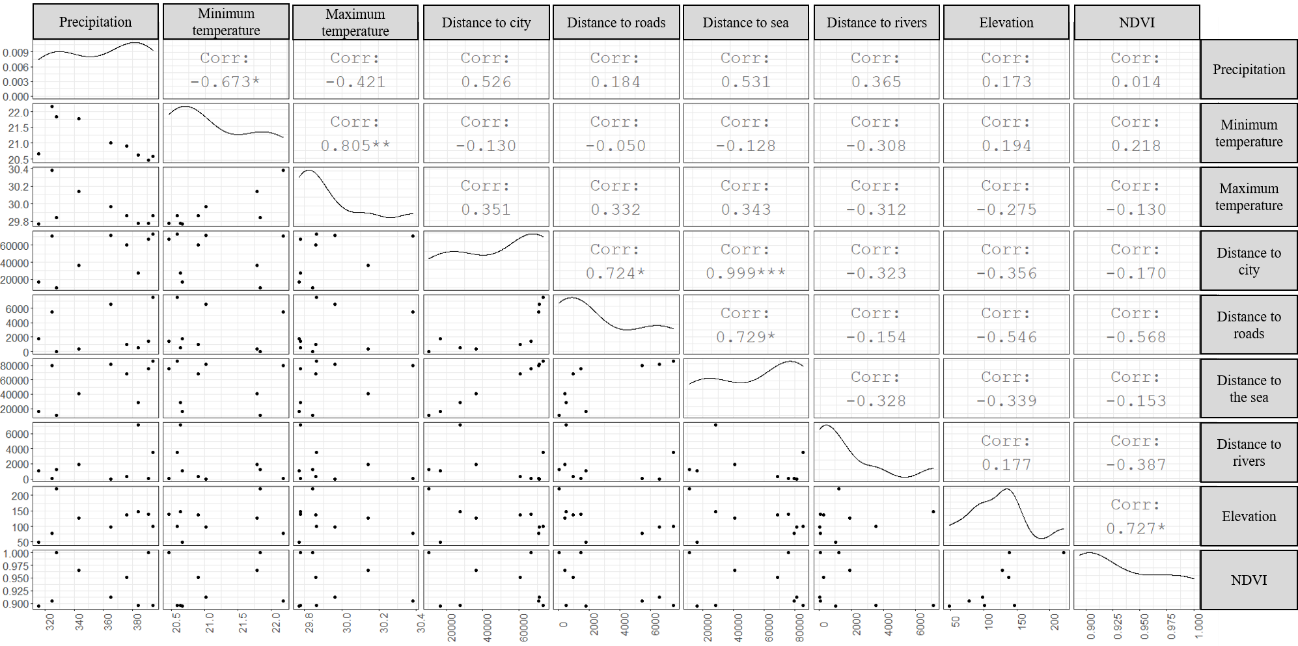
 **Figure S4. Correlation plot showing the results for Pearson’s correlation test on abiotic variables that are relevant for haemosporidian prevalence distribution.** Significant results are marked with an asterisk (*).

**
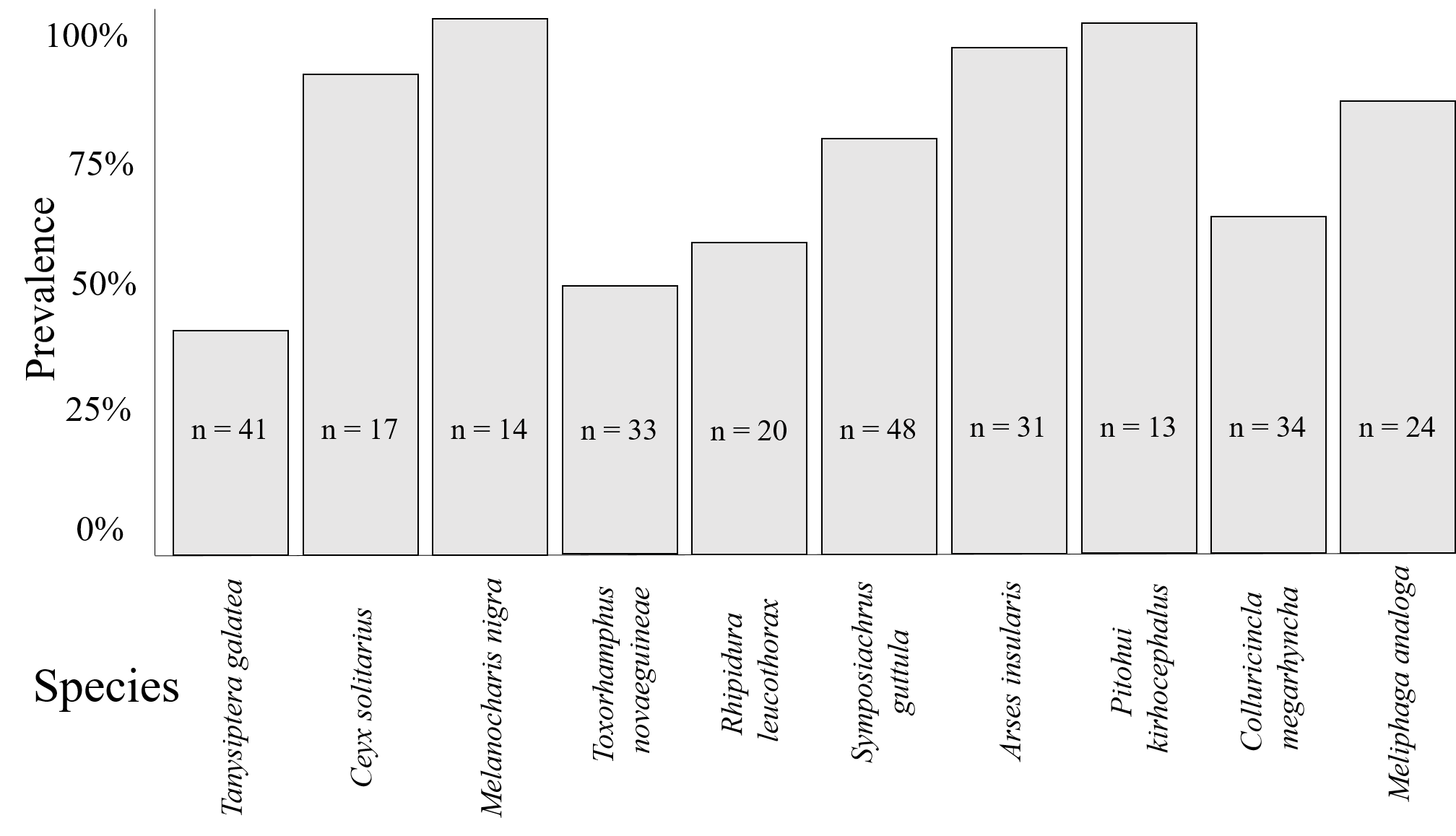
Figure S5. Total Haemosporidian prevalence (%) per species in 2015**. Numbers of sampled individuals (n) are shown inside the bars.

**
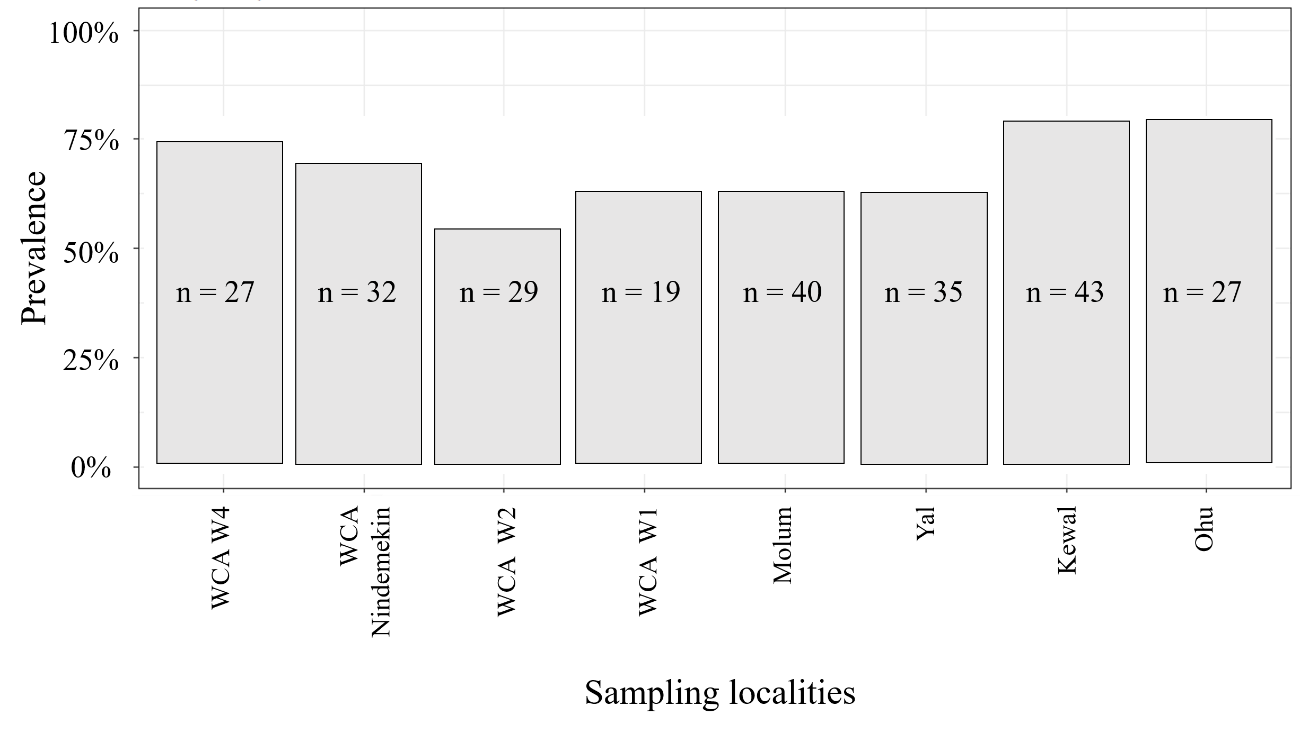
Figure S6.** ***Haemoproteus* prevalence (%) in the different localities sampled in 2015.** Numbers of sampled individuals (n) are shown inside the bars. Localities are ordered by distance to the sea; closest to the right and furthest to the left.


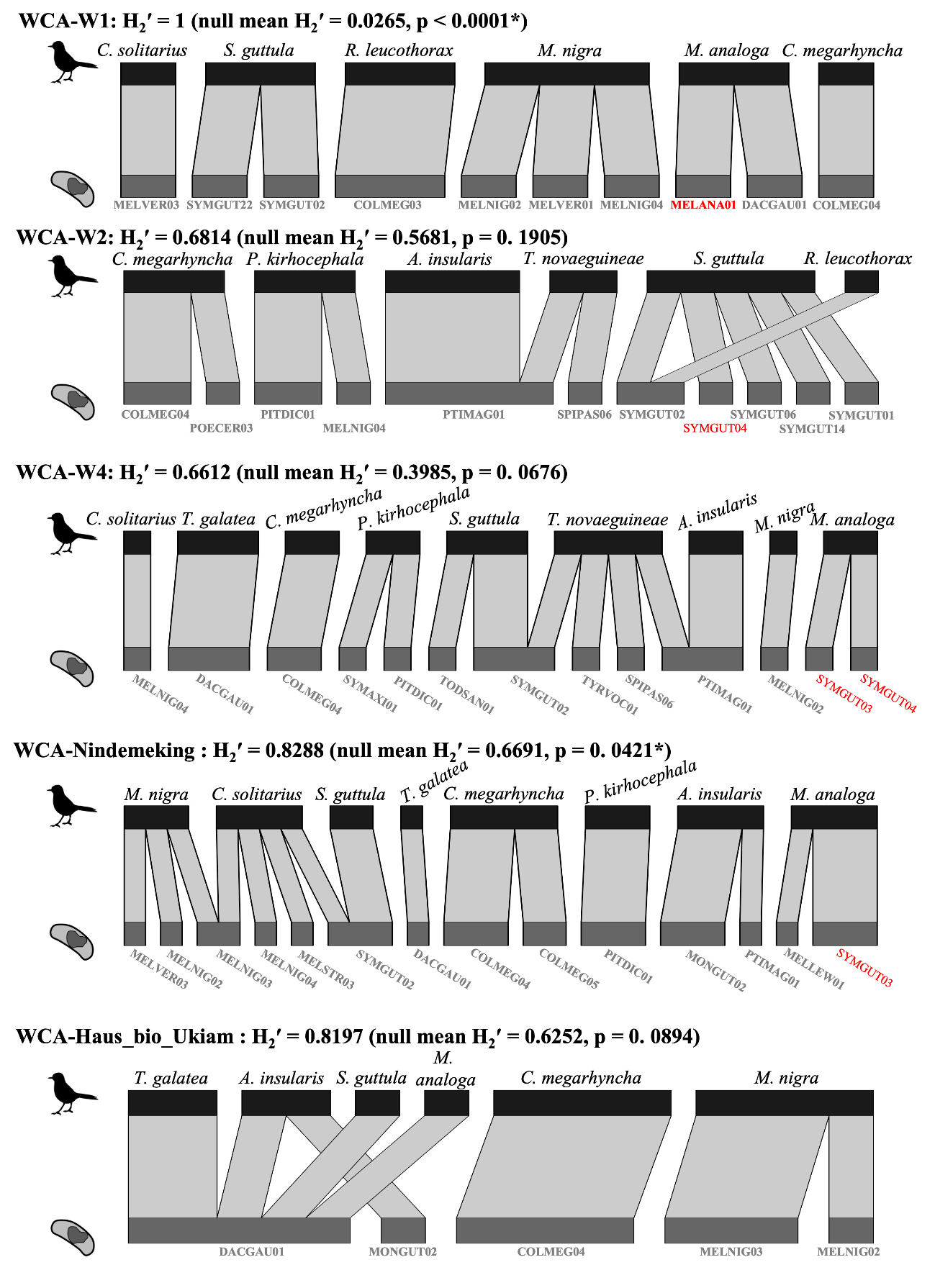
 **Figure S7. Bird-haemosporidian networks in each of the localities within the continuous Wanang forest.** Observed H_2_′ and the average H_2_′ acquired from 1,000 null models are given within parenthesis. Significantly different H_2_′ compared to expected by chance are indicated with asterisks. Bird species names in black, *Haemoproteus* lineage names in grey, and *Plasmodium* lineage names in red. An H2′ index closer to 1 indicates that host–parasitic communities are more specialized (many one-to-one associations), while highly generalized networks have H2′ indices closer to 0.


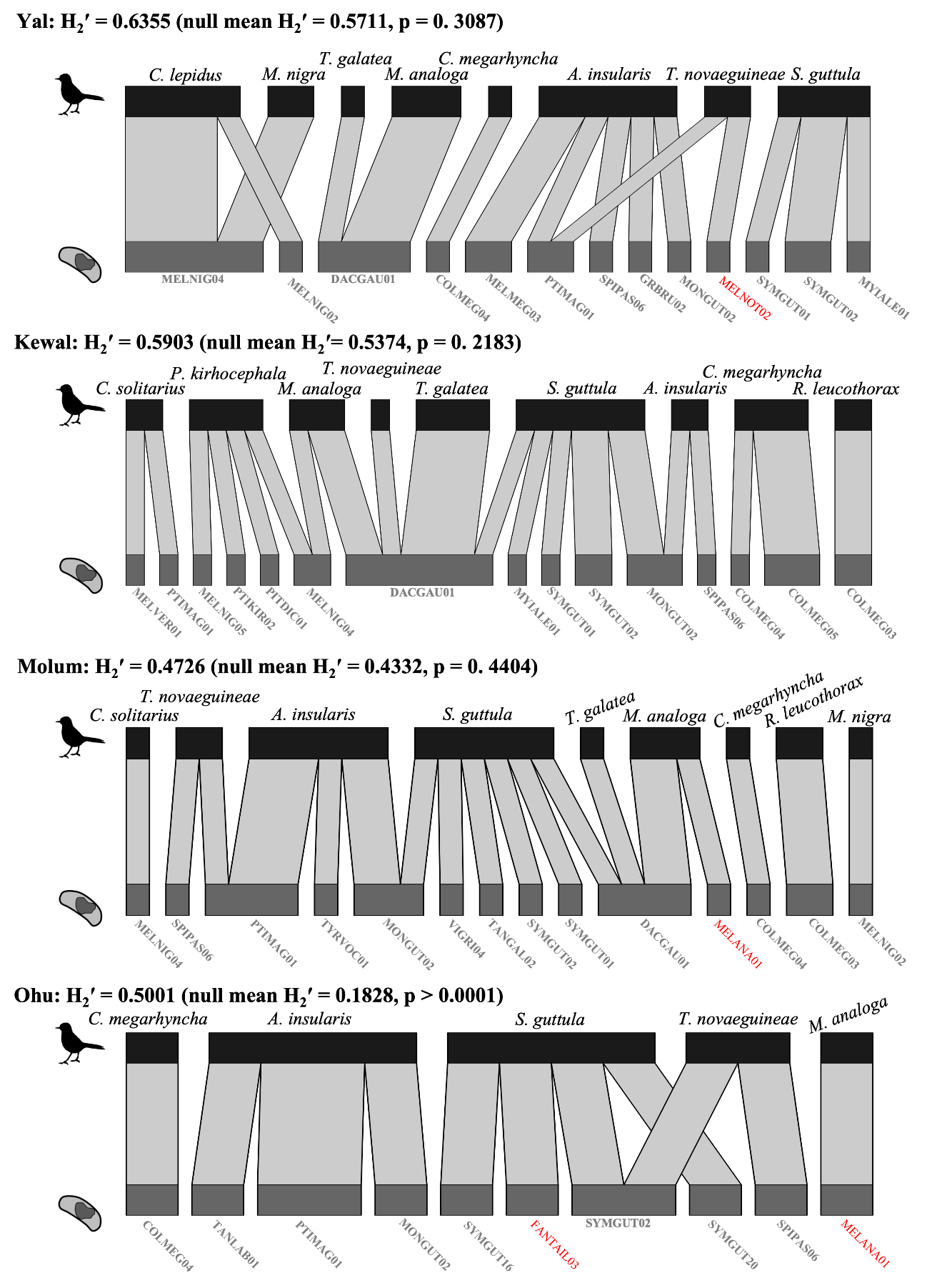
 **Figure S8. Bird-haemosporidian networks in different lowland forest fragments.** Observed network-level specificity index (H2′) and the average H2′ from 1,000 null models are given within parentheses. Bird species names in black, *Haemoproteus* lineage names in grey, and Plasmodium lineage names in red. An H2′ index closer to 1 indicates that host–parasitic communities are more specialized (many one-to-one associations), while highly generalized networks have H2′ indices closer to 0.


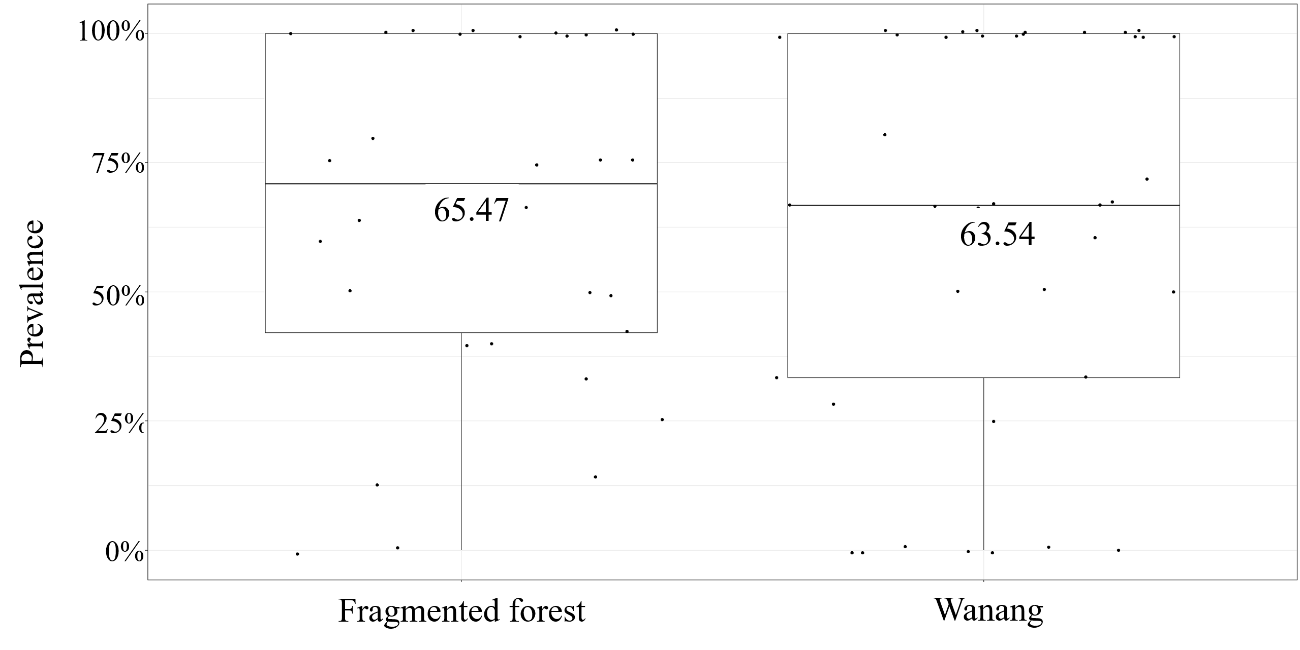
**Figure S9. Box plots depict mean prevalences (± SD) of bird species from 2015 in the fragmented forest and the Wanang area (continuous forest).** Dots indicate individual observations of prevalence by species and numbers inside the boxplots are the mean prevalence values. Prevalence did not significantly differ between continuous and fragmented forest.

**
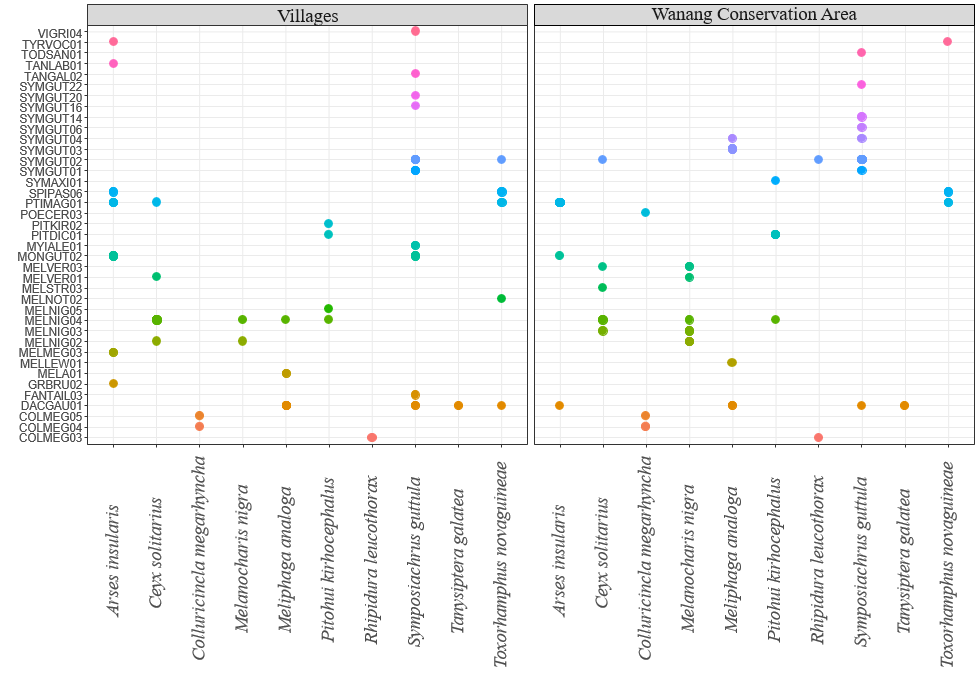
 Figure S10. Parasite lineages found in the continuous forest (all the localities within Wanang Conservation Area) and the fragmented forest (all the fragmented forests).** Dots indicate the presence of a parasite lineage in a species. There are thirteen shared lineages: TYRVOC01, SYMGUT02, SYMGUT01, SPIPAS06, PTIMAG01, MONGUT02, MELVER01, MELNIG04, MELNIG02, DACGAU01, COLMEG04, COLMEG05 and COLMEG05.

**
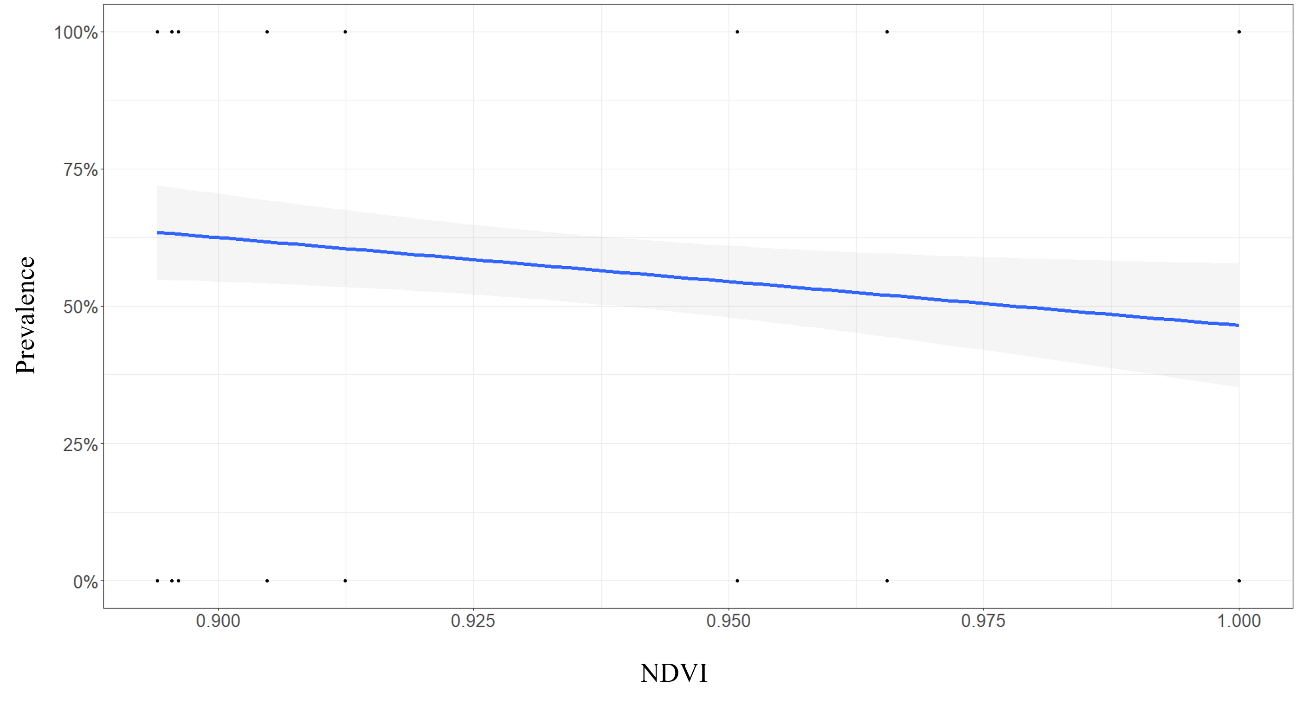
**

**Figure S11. Associations of *Haemoproteus* prevalence NDVI across all sampling sites sampled in 2015.** Dots represent infected (100%) or non-infected (0%) individuals and lines (linear model) show the prevalence changes associated with each variable.


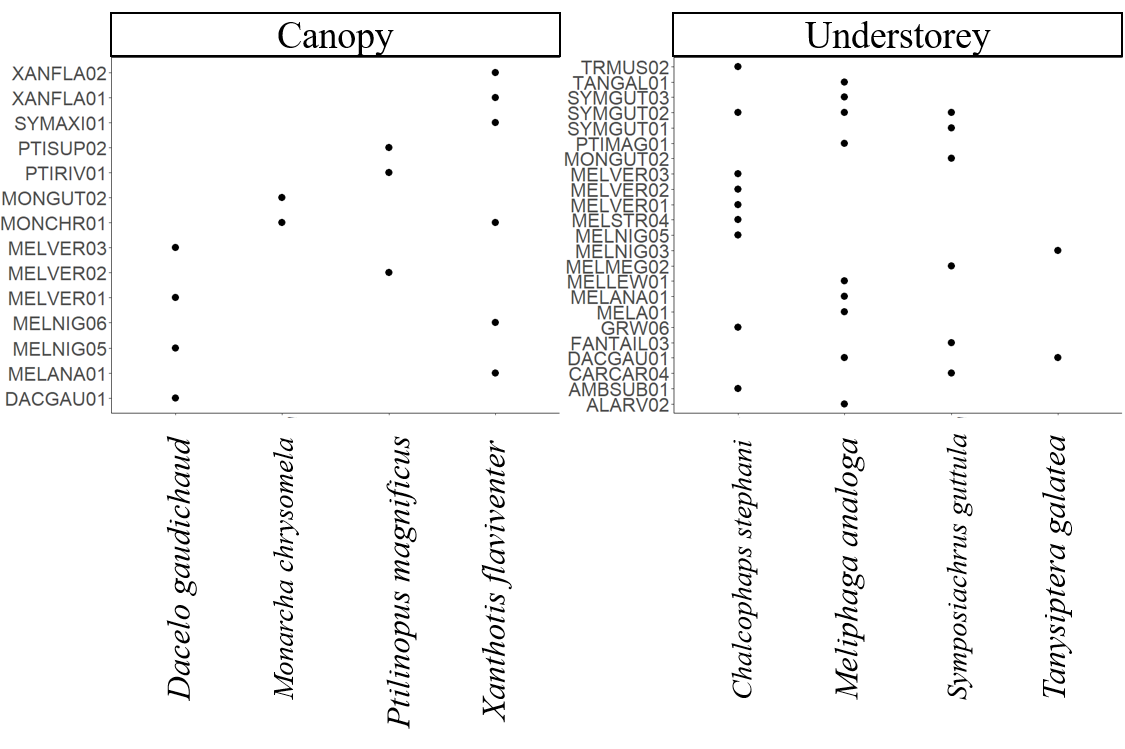
**Figure S12. Parasite lineages found in the canopy and the understorey.** Dots indicate the presence of a parasite lineage in a species. There are seven parasite lineages shared between the understory and the canopy: MONGUT02, MELVER03, MELVER02, MELVER01, MELNIG05, MELANA01 and DACGAU01.

**Figure S13. Host-parasite network structures and the haemosporidian lineage level specificity in canopy and understory bird species at Wanang 3.** A) Four canopy bird species and B) four understorey bird species from the same families were investigated in this analysis. Observed network specificity index (H2′) and the average H2′ from 1,000 null models are given within parentheses. Bird species names in black, *Haemoproteus* lineage names in grey, and *Plasmodium* lineage names in red. An H2′ index closer to 1 indicates that host–parasitic communities are more specialized (many one-to-one associations), while highly generalized networks have H2′ indices closer to 0. Asterisks represent haemosporidian lineages found in both canopy and understorey species. C) Rao’s quadratic entropy (Host specificity of lineages) of most frequent parasite lineages (for lineages that have infected more than two bird individuals) demonstrated more specialist lineages in the understorey compared to canopy.
